# Prevalence of Appropriate Anatomic Total Shoulder Arthroplasty in a Large Multicenter US Cohort Using a RAND/UCLA Algorithm

**DOI:** 10.5435/JAAOSGlobal-D-24-00366

**Published:** 2025-11-12

**Authors:** Krishna Mandalia, Stephen Le Breton, Christopher Roche, Katharine Ives, Sarav Shah

**Affiliations:** From the New England Baptist Hospital, Boston, MA (Mandalia, Le Breton, Dr. Shah); the Tufts University School of Medicine, Boston, MA (Mandalia, Le Breton); the New England Shoulder and Elbow Center, Boston, MA (Mandalia, Le Breton, Ives, Dr. Shah); and the Exactech Inc, Gainesville, FL (Roche).

## Abstract

**Background:**

Given the high variability in patient presentation, notable challenges exist in determining patient candidacy for anatomic total shoulder arthroplasty (aTSA). The purpose of this study was to use a modified version of prior scenario-based appropriateness use criteria to evaluate the prevalence of inappropriate, appropriate, and inconclusive aTSA.

**Methods:**

Patients undergoing primary aTSA were evaluated for preoperative outcome scores and baseline demographic information from a multicenter database. Using a validated appropriateness use criteria algorithm, these patients were grouped “inappropriate,” “inconclusive,” or “appropriate.”

**Results:**

Seven hundred seventy-four patients who underwent aTSA were included. “Appropriate” patients comprised 23.9% of the cohort, while 17.8% were “inappropriate,” and 58.3% were “inconclusive.” Compared with the inconclusive and inappropriate groups, the “appropriate” patients were found to have markedly worse preoperative pain and functional outcomes scores. No notable difference was observed between the number of patients who received intra-articular injections, number of injections received, and analgesic use across the groups.

**Conclusions:**

The large proportion of “inconclusive” patients suggests a lack of consensus regarding aTSA versus reverse TSA candidacy and may be secondary to factors such as worse glenoid morphology and/or prior rotator cuff repair, which are subjects of current debate in determining appropriateness for reverse TSA versus aTSA. Although no definitive conclusions can be made regarding if this algorithm ultimately improves patient outcomes, this study seeks to only help streamline patient evaluation based on American Shoulder and Elbow Surgeons high-volume surgeons' opinion and highlight the large variation in the indications for aTSA in real-world surgical cases.

Numerous contemporary publications have described the remarkable growth in the use of anatomic total shoulder arthroplasty (aTSA) in the United States.^1-3^ Total shoulder arthroplasty is a method of treating primary glenohumeral osteoarthritis that has consistently demonstrated acceptable medium-term and long-term outcomes.^4,5^ However, notable variability in patient presentation can lead to challenges with clinical decision making, especially in patients who may be nonsurgical candidates or better suited for other procedures (e.g., reverse TSA due to cuff tear arthropathy and irreparable rotator cuff tears).^1,2,6,7^ Until recently, there has been an absence of evidence-based guidelines to assist surgeons in determining patient-eligibility for aTSA. A recent study by Le Breton et al^1^ used the RAND/University of California at Los Angeles (UCLA) method to create and validate appropriateness use criteria for primary anatomic TSA. The UCLA/RAND method is a tool to improve quality of care, evaluate appropriateness, control costs, and support clinical decision making in the absence of well-defined guidelines.^8^ It has been previously used to evaluate appropriateness for a number of orthopaedic interventions.^1,9,10,11,12,13,14^ After generation of these criteria, patient data can be used to characterize appropriate patient populations, calculate overuse or underuse of a procedure, or determine timeliness of the procedure in appropriate patients.^13,15-17^

The study by Le Breton et al^1^ successfully used a Delphi method approach with two independent panels composed of 12 high-volume American Shoulder and Elbow Surgeons (ASES) surgeons, as well as a robust review of current literature, to construct a clinical decision-making tree for appropriateness of anatomic TSA. The study found that ASES surgeon panel members agreed on ∼64% of indications and that ∼24% of studied hypothetical clinical scenarios were deemed appropriate for primary anatomic TSA. Notably, a notable number of scenarios were deemed inconclusive, highlighting the lack of consensus on various patient characteristics in determining primary anatomic TSA candidacy. Age, symptomatology, and Walch classification were identified as key variables that influenced patient appropriateness for aTSA.

The increased utilization of aTSA calls for appropriateness criteria that has the capability to enhance quality of care and control costs, as well helping to guide physician-patient shared decision making. Currently, no clinical studies have investigated aTSA patients with the purpose of stratifying and characterizing them into appropriateness classification groups. Thus, the purpose of this study was to determine the prevalence of appropriate and inappropriate anatomic TSAs performed using a modified form of a validated appropriateness use criteria algorithm in a multicenter national cohort and to secondarily report the baseline, preoperative characteristics of patients within each group. We hypothesize a higher proportion of “inconclusive” group patients compared with “appropriate” and “inappropriate” group patients given the developing consensus and ongoing debate among orthopaedic surgeons regarding indications for anatomic TSA versus reverse TSA in the setting of glenohumeral arthritis. We also hypothesize that those patients deemed “appropriate” by our algorithm will have more severe preoperative pain and functional limitations, abetting in the clinical decision making to undergo anatomic TSA.

## Methods

### Subject Inclusion and the Database

Subjects were derived from a multicenter database of 3833 aTSA patients for whom a single-platform prosthesis was used, between November 2004 and October 2021. Every patient enrolled in the database provided consent; data were collected using standardized forms and scored on a secure IBM database. Surgery was done across 30 clinical sites. The database provides comprehensive patient demographic characteristics (e.g., age, ethnicity, BMI, implant type, comorbidities) as well as preoperative objective and patient-reported outcome measures such as the Visual Analog Pain Scale (VAS) score, Simple Shoulder Test score, ASES score, UCLA Shoulder score, Pain and Disability Index score, Constant score, and Shoulder Arthroplasty Smart (SAS) score.

Patients who had undergone primary anatomic TSA and who had preoperative information that detailed age, mobility (active ROM for abduction, forward flexion, internal rotation, and external rotation), symptomatology (preoperative VAS Pain Scale), rotator cuff status, and CT (Walch classification) were included. Patients were also required to have at least one preoperative patient-reported outcome measure (Constant, Simple Shoulder Test, ASES, Shoulder Pain and Disability Index, UCLA, and/or SAS) reported. Patients who had undergone revision TSA, reverse TSA, or hemiarthroplasty, as well as TSA-eligible patients who did not undergo surgery, were excluded. This resulted in inclusion of 774 patients.

### Classification Criteria

This study leveraged a modified version of the appropriateness classification criteria described by Le Breton et al.^1^ The criteria for age (>75 years, <65 years, and 65 to 75 years), mobility (preserved mobility requires active range of motion of 140° of abduction, >140° of forward flexion, internal rotation to the L4 spinous process or higher, and 40° of external rotation), and Walch classification were the same as the previous study.^1^ Rotator cuff status simplified to focus on if patients had an intact rotator cuff versus prior rotator cuff surgery and/or current full-thickness tear based on patient chart review, physical examination, and imaging studies. Symptomatology was simplified to use validated VAS Pain scale cutoffs for slight (score ≤ 4), moderate (score 5 to 6), and severe (score ≥ 7) symptomatology because other requirements (e.g., Activities of Daily Living (ADLs) and level of pain control with analgesia) were not available in the database.^18^ Preoperative Samilson & Prieto (SP) classification was also not available in this database. This is a limitation but given SP classification was demonstrated in our previous study to have minimal effect on variability in appropriateness categorization, we elected to remove the criterion from the appropriateness algorithm entirely.^1^ Equivalent scenarios across the three SP classifications were combined into a single scenario, and appropriateness of the combined scenario was determined using the average of appropriateness scores from the three original scenarios (e.g., three scenarios with scores of 2, 2, and 3 would average to 2.33, which qualifies as inappropriate).

### Data Analysis

**Figure 1:**
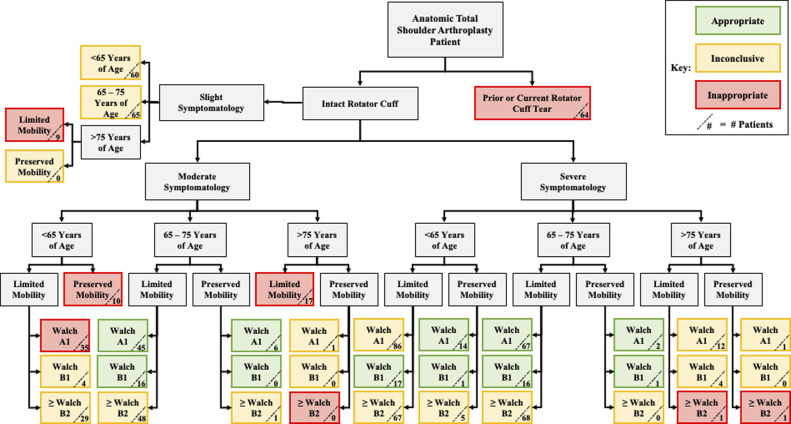
Diagram showing the TSA patient distribution across symptomatology indications. Reproduced, with modification, from: [Le Breton et al^1^]. Reproduced with permission. TSA = total shoulder arthroplasty.

Appropriateness classifications were determined using the modified algorithm described above (Figure 1).^1^ Statistics were conducted using IBM Statistical Package for Social Sciences Statistics (IBM). Statistical significance of differences between appropriateness groups was determined using Pearson chi-square for categorical variables. One-way ANOVA with Tukey post hoc was used for continuous variables.

## Results

### Prevalence of Appropriateness Categories

Seven hundred seventy-four patients who underwent primary anatomic TSA were included in the study; 185 (23.9%) were considered appropriate, 451 (58.3%) as inconclusive, and 138 (17.8%) as inappropriate. The average age at surgery was 64.4 years among the entire cohort. Age at time of surgery was significantly higher in the appropriate group (66.8 ± 6.1) compared with the inconclusive (63.5 ± 7.4) and inappropriate (63.9 ± 10.8) groups (*P* < 0.001). Forty-three percent of the entire cohort was female. The appropriate group had a significantly less proportion of patients who were male (49%) compared with the inconclusive (59%) and inappropriate (62%) groups (*P* = 0.027). The average BMI of the cohort was 30.2. The appropriate group (29.4 ± 6.2) had a significantly lower BMI than the inconclusive group (30.7 ± 6.2, *P* = 0.030). Ninty-two percent of the entire cohort was White. Seventy-one percent of patients had a medical comorbidity of which hypertension was the most common (51%). The inappropriate group (43%) had a significantly less proportion of patients with hypertension compared with appropriate (57%) and inconclusive (50%) groups (*P* = 0.026). No significant difference was observed in race, or most medical comorbidities (with the exception of hypertension) across appropriateness classification groups (Table 1).

**Table 1:** Demographics and Preoperative Outcome Scores

Algorithm Variables	All Patients (N = 774)	Appropriate (N = 185)	Inconclusive (N = 451)	Inappropriate (N = 138)	Significance
Age at surgery, years (mean ± SD)	64.4 ± 7.9	66.8 ± 6.1	63.5 ± 7.4	63.9 ± 10.8	*P* < 0.001^a^
Body mass index (mean ± SD)	30.2 ± 6.2	29.4 ± 6.2	30.7 ± 6.2	29.7 ± 6.1	*P* = 0.030^b^
Days hospitalized (mean ± SD)	0.89 ± 0.72	1.0 ± 0.8	0.83 ± 0.63	0.95 ± 0.86	*P* = 0.024^b^
Sex					*P* = 0.027
Male (N, %)	445 (57)	91 (49)	268 (59)	86 (62)	
Female (N, %)	329 (43)	94 (51)	183 (41)	52 (38)	
Race					*P* = 0.799
White (N, %)	714 (92)	168 (91)	420 (93)	126 (91)	
African American (N, %)	34 (4)	9 (5)	18 (4)	7 (5)	
Hispanic (N, %)	9 (1)	3 (2)	3 (1)	3 (2)	
Asian (N, %)	5 (1)	2 (1)	2 (0)	1 (1)	
Not available (N, %)	12 (2)	3 (2)	8 (2)	1 (1)	
Comorbidities					
Any comorbidities (N, %)	549 (71)	140 (76)	316 (70)	93 (67)	*P* = 0.118
Inflammatory arthritis (N, %)	54 (7)	9 (5)	34 (8)	11 (8)	*P* = 0.451
Hypertension (N, %)	391 (51)	106 (57)	225 (50)	60 (43)	*P* = 0.026^c^
Heart disease (N, %)	93 (12)	28 (15)	48 (11)	17 (12)	*P* = 0.262
Diabetes (N, %)	86 (11)	25 (14)	50 (11)	11 (8)	*P* = 0.262
Tobacco use (N, %)	54 (7)	10 (5)	32 (7)	12 (9)	*P* = 0.543
Tobacco pack years (mean ± SD)	15.4 ± 18.5	14.1 ± 19.3	15.7 ± 18.9	16.1 ± 17.5	*P* = 0.935
Chronic renal failure (N, %)	7 (1)	3 (2)	2 (0)	2 (1)	*P* = 0.269
Other comorbidities (N, %)	196 (25)	52 (28)	114 (25)	30 (22)	*P* = 0.690
Injections					
Received any injection (N, %)	415 (54)	98 (53)	237 (53)	80 (58)	*P* = 0.525
Average number of corticosteroid injections (mean ± SD)	2.4 ± 5.5	3.3 ± 10.8	2.0 ± 1.7	2.3 ± 2.6	*P* = 0.205
Average number of viscosupplementation injections (mean ± SD)	0.3 ± 0.9	0.1 ± 0.3	0.5 ± 1.3	0.1 ± 0.4	*P* = 0.182
Analgesics					
Used any analgesic (N, %)	473 (61)	109 (59)	283 (63)	81 (59)	*P* = 0.543
NSAIDs (N, %)	220 (28)	55 (30)	131 (29)	34 (24)	*P* = 0.545
Opioids (N, %)	74 (10)	18 (10)	42 (9)	14 (9)	*P* = 0.955
Other (N, %)	30 (4)	15 (7)	12 (2)	3 (2)	*P* = 0.003^c^
Not reported (N, %)	194 (25)	41 (22)	116 (26)	37 (27)	*P* = 0.561
Device type					*P* = 0.829
Standard total (N, %)	278 (36)	68 (37)	159 (35)	51 (37)	
Standard total with preserved stem (N, %)	263 (34)	64 (34)	149 (33)	50 (36)	
Standard total with stemless humeral implant (N, %)	233 (30)	53 (29)	143 (32)	37 (27)	
Preoperative outcome measures					
VAS Pain Scale (mean ± SD; n = 774)	6.3 ± 2.1	7.2 ± 1.5	6.2 ± 2.4	5.5 ± 1.6	*P* < 0.001^d^
SST (mean ± SD; n = 433)	4.6 ± 2.8	4.5 ± 2.5	4.6 ± 3.0	4.9 ± 2.8	*P* = 0.547
Constant score (mean ± SD; n = 444)	41.8 ± 15.5	41.6 ± 15.3	40.8 ± 15.4	45.4 ± 15.5	*P* = 0.058^e^
ASES (mean ± SD; n = 761)	38.7 ± 15.8	33.7 ± 12.7	39.3 ± 17.1	43.3 ± 13.1	*P* < 0.001^d^
UCLA (mean ± SD; n = 454)	15.5 ± 4.1	15.2 ± 3.2	15.5 ± 4.5	15.9 ± 4.0	*P* = 0.490
SPADI (mean ± SD; n = 461)	77.7 ± 21.9	81.2 ± 17.9	77.2 ± 23.6	74.7 ± 20.7	*P* = 0.097
SAS (mean ± SD; n = 770)	48.8 ± 11.9	48.17 ± 11.0	48.3 ± 12.4	51.4 ± 11.3	*P* = 0.019^c^

a Statistically significant difference between appropriate and inconclusive, and appropriate and inappropriate.

b Statistically significant difference between appropriate and inconclusive.

c Statistically significant difference between inappropriate and appropriate, and inappropriate and inconclusive.

d Statistically significant differences present between all three groups.

e Statistically significant difference between inappropriate and inconclusive (*P* = 0.046).

ASES = American Shoulder and Elbow Surgeons, SAS = Shoulder Arthroplasty Smart, SPADI = Shoulder Pain and Disability Index, SST = Simple Shoulder Test, VAS = Visual Analog Pain Scale

A total of 473 patients (61%) reported using any analgesic; Non-Steroidal Anti-Inflammatory Drugs (NSAIDs) (28%) were the most frequently used analgesic. Four hundred fifteen patients (54%) reported receiving any intra-articular injection before aTSA, with a mean of 2.4 corticosteroid injections and 0.3 viscosupplementation injections per patient across the study population. No notable difference was observed between the number of patients who received intra-articular injections and the number of injections received. Similarly, there was no notable difference in analgesic use across the appropriateness classification groups. No notable difference was also observed between device-type across groups.

### Characterization of Patients Within Each Appropriateness Category

A total of 185 of the 774 subjects (23.9%) were classified appropriate (Figure 1). A significantly higher proportion of appropriate patients (64%) demonstrated severe symptomatology relative to inconclusive or inappropriate patients (Table 2). The more severe symptomatology was also reflected by preoperative outcome scores, with appropriate patients demonstrating significantly worse preoperative scores on VAS, ASES, and SAS (Table 1). In addition, appropriate patients were of most commonly between 65 and 75 years (83%) and were markedly more likely to have hypertension (57%) relative to inappropriate patients. No appropriate patients had a Walch classification ≥ B2 or were of older than 75 years. Of note, appropriate patients had a statistically notable longer hospital stay (1.0 days) than inconclusive (0.83 days), but not inappropriate patients (0.95 days).

**Table 2:** Patient Characteristics Based on Appropriateness Algorithm Criteria

Algorithm Variables	All Patients (N = 774)	Appropriate (N = 185)	Inconclusive (N = 451)	Inappropriate (N = 138)	Significance
Rotator cuff status					*P* < 0.001^a^
Intact rotator cuff (N, %)	710 (92)	185 (100)	451 (100)	74 (54)	
Prior RTC surgery/current full-thickness tear (N, %)	64 (8)	0 (0)	0 (0)	64 (46)	
Symptomatology					*P* < 0.001^b^
Slight (N, %)	151 (20)	0 (0)	125 (28)	26 (19)	
Moderate (N, %)	233 (30)	67 (36)	83 (18)	83 (60)	
Severe (N, %)	390 (50)	118 (64)	243 (54)	29 (21)	
Age group					*P* < 0.001^b^
<65 years (N, %)	370 (48)	32 (17)	251 (56)	87 (63)	
65–75 years (N, %)	352 (45)	153 (83)	182 (40)	17 (12)	
>75 years (N, %)	52 (7)	0 (0)	18 (4)	34 (25)	
Mobility					*P* = 0.007^b^
Limited (N, %)	707 (91)	161 (87)	424 (94)	122 (88)	
Preserved (N, %)	67 (9)	24 (13)	27 (6)	16 (12)	
Walch classification					*P* < 0.001^b^
A1 (N, %)	379 (49)	134 (72)	160 (35)	85 (62)	
B1 (N, %)	82 (11)	51 (28)	22 (5)	9 (7)	
≥B2 (N, %)	313 (40)	0 (0)	269 (60)	44 (32)	

RTC = Rotator Cuff tear

a Pearson chi-square demonstrates a statistically significant difference between the proportion of patients with “any prior RTC surgery” across groups. Of note, this is expected as patients with any prior RTC surgery were classified “inappropriate.”

b Chi-square demonstrates a statistically significant difference between groups.

Most of the patients undergoing aTSA (58.3%) were classified inconclusive. Inconclusive patients were markedly more likely to have slight or moderate symptomatology than appropriate patients, although 54% of inconclusive patients still demonstrated severe symptomatology. Inconclusive patients demonstrated markedly higher VAS and lower ASES scores versus inappropriate patients, but lower VAS and higher ASES scores versus appropriate patients. They were also more likely to be younger than appropriate patients, with 56% of inconclusive patients undergoing aTSA at younger than 65 years. They were also more likely to have glenoid morphology of Walch ≥ B2 compared with appropriate patients.

Approximately 138 (17.8%) of the 774 patients undergoing aTSA were classified inappropriate. Sixty-four inappropriate patients (46%) were classified inappropriate due to either a history of rotator cuff surgery or new rotator cuff tear found during surgery. Most inappropriate patients demonstrated moderate symptomatology (60%) as well as markedly better preoperative VAS and ASES scores versus appropriate and inconclusive patients. In addition, inappropriate patients exhibited markedly better preoperative SAS scores relative to appropriate and inconclusive patients. Inappropriate patients were found to have a bimodal age distribution of either younger than 65 years (63%) and older than 75 years (25%). Similar to inconclusive patients, inappropriate patients were more likely to have Walch ≥ B2 than appropriate patients.

## Discussion

Using a validated appropriateness use criteria with modifications, we found that approximately 18% of primary anatomic TSAs performed in a large US-based multicenter cohort were classified inappropriate. Fundamentally, the RAND/UCLA method used by Le Breton et al^1^ is intended for approximations of appropriateness of aTSA in groups and is not intended for use with individual patients. Although many patients were classified “inconclusive,” which is a reflection of poor consensus of patient characteristics regarding aTSA candidacy, we endorse the use of these criteria/algorithm because it may benefit lower-volume surgeons and facilities in understanding and guiding shared decision making of treatment options in the setting of glenohumeral arthritis. Given this high variability in patient presentation, our study presents findings that may benefit the clinical decision-making process regarding aTSA candidacy.

One finding of this study that may be controversial was the proportion of patients classified inappropriate for primary anatomic TSA (17.8%). Approximately 64 of the 138 patients (46%) classified “inappropriate” were driven by a history of rotator cuff repair (RCR). Previous studies have demonstrated that preserved rotator cuff function after aTSA is critical to patient outcomes.^7,19-21^ As such, the pendulum has swung toward use of reverse shoulder arthroplasty (RSA) as opposed to anatomic TSA in patients where there is concern of rotator cuff dysfunction (e.g., partial tear and history of prior repair). RSA has a strong clinical performance in patients with a diagnosis of cuff tear arthropathy and has become the primary treatment modality for those with cuff arthropathy.^22^ However, classification of all patients with prior RCR as inappropriate for aTSA may be a source of contention because aTSA after RCR may result in similar improvements in shoulder function compared with those without prior surgery despite a higher risk of postoperative complications.^23^ In addition, improvements in MRI allow for preoperative screening of fatty infiltration or partial retear, providing greater confidence in an intact rotator cuff status in patients with a remote history of RCR.^24,25^ Thus, this may be an overestimation of inappropriate patients because patients may have a remote history of successful RCR. Furthermore, our prospectively collected registry did not track time between RCR and anatomic TSA.

In addition, inappropriate patients typically were characterized by better preoperative outcome measures and were either younger than 65 years with slight or moderate symptomatology or older than 75 years.^1^ Inappropriateness in patients >75 years is likely driven by national trends that support use of RSA. However, new evidence by Shah et al.^21^ suggests a higher percentage of anatomic TSA patients achieved substantial clinical benefit versus patients undergoing RSA at 2-year follow-up. In that study, for patients aged 75 years or older, there was a similar percentage of patients who achieved the minimal clinically important difference (aTSA, 93.1%; RTSA, 92.3%; *P* = 0.53); however, a greater proportion of anatomic TSA patients achieved substantial clinical benefit (90.5% vs 76.9%; *P* = 0.01). Inappropriateness in younger patients with slight or moderate symptomatology was likely driven by concerns regarding need for future revision and manageability of pain with nonsurgical intervention (e.g., analgesia and intra-articular corticosteroid injections).^1^ However, better implant survivability and improved ability to convert to RSA may provide greater rationale for use in younger patients.^2,26,27^ Nonetheless, these differences in approach to treatment of glenohumeral arthritis suggest the need for additional investigation of patient characteristics and their influence on aTSA candidacy.

A notable percentage of patients were grouped inconclusive (58.3%), which reflects the disagreement among high-volume members of ASES present in the pilot study by Le Breton et al.^1^ Inconclusive patients tended to be younger and have less severe symptomatology than appropriate patients. Similar to inappropriate patients, inconclusiveness in younger patients was driven by concerns regarding need for future revision and preference for nonsurgical pain management. In addition, both inappropriate and inconclusive patients were more likely to have worsening glenoid morphology (i.e., Walch classification ≥B2). This finding may reflect bias from the previous study given no patients with a Walch ≥B2 were classified appropriate but is also consistent with existing evidence that suggests patients with Walch ≥ B2 are more likely to have worse outcomes with aTSA, and are therefore better suited for reverse shoulder arthroplasty.^28^ Given the lack of clear guidelines and consensus regarding these scenarios (i.e., young patient with Walch ≥B2 glenoid morphology), most of the patients included were categorized inconclusive, limiting the applicability of the appropriateness use criteria/algorithm.

Utilization of the aTSA appropriateness algorithm is based on the concept that primary anatomic TSA should be conducted on patients who have severe symptomatology, but are not of an age or have severe enough glenoid morphology where they would instead require reverse TSA. Patients with severe symptomatology and/or severe radiographic findings are likely to show greater benefit from surgical intervention due to greater baseline deficits.^29^ While logical, this line of clinical reasoning may fail to consider that patients who are less symptomatic, and thus may be considered inappropriate for aTSA at that time, may benefit from earlier surgery to prevent an extended period of morbidity and reduced function. For such patients, it may be appropriate to undergo anatomic TSA at an earlier stage, rather than wait for symptomatology to progress. This point of debate is demonstrated in the notable number of inconclusive patients identified by this study and reflects the strong controversy surrounding existing evidence for primary anatomic TSA. This concept of “delayed benefit” is especially salient in the setting of new evidence that demonstrates increased implant survivability and improved convertibility to reverse TSA.^26,27,30^ As such, improved implant life span and convertibility to RSA may overestimate the true proportion of inconclusive or inappropriate patients.

As with any study, there are limitations. Our study included 774 (20.1%) of a cumulative database of 3833 total shoulder arthroplasty patients. Unfortunately, the study sample was limited secondary to the strict inclusion criteria, including both the exclusion of nonanatomic shoulder arthroplasties and requirement for each subject to have all preoperative datapoints included in the modified classification criteria. In the authors' opinion, this may have pushed the data set away from the null hypothesis toward a higher percentage of inappropriate classification of patients. Moreover, our cohort spans 2004 to 2021 during which time the growth of RSA materialized and implants changed. Although our cohort implemented a single-implant platform, modifications and changes were still made over that period. Thus, given the increased debate and improvement in the RSA, the usage of the anatomic prosthesis may have declined with a converse increase in RSA; thus, more and more surgeries fell under the “inconclusive” and “inappropriate” groups over time because options for patients with cuff disease and/or worsened bone loss, retroversion, etc. evolved. To that matter, the database was deidentified, and thus, patient name, date of birth, and treatment dates were unavailable. Subsequently, we were unable to perform time-based analyses (i.e. year-on-year change in proportion of aTSA and RSA). Furthermore, modifications were made to the appropriate use classification criteria set by the previous study. These were deemed necessary to adjust for differences between data available for review and the original hypothetical clinical scenario-based appropriateness classification scheme. Samilson and Prieto (SP) classification unfortunately was not collected in our prospectively collected registry; thus, the appropriateness algorithm was modified to exclude SP classification. Joyce et al^31^ found that no clinically influential associations were found between SP Grade 4 and ASES score. In addition, Le Breton et al.^1^ found that SP classification had the lowest influence on variability among appropriateness groups. Similarly, symptomatology was modified to best correlate with available data because activities of daily living and level of pain control with analgesia were not available. VAS Pain Scale cutoffs for slight, moderate, and severe symptomatology were adapted using cutoffs from prior published literature evaluating chronic musculoskeletal pain.^18^ Thus, the authors think that the variations made were negligible because adept modifications were made to hold the essence of the appropriate use criteria. Finally, outcomes data were not evaluated in this study, and thus, appropriateness groups were not evaluated to characterize difference in postoperative outcomes. Therefore, no definitive conclusions can be made to determine whether the use of this algorithm will ultimately improve patient outcomes after anatomic TSA. To that matter, this study seeks to only help streamline patient evaluation based on ASES high-volume surgeons' opinion and highlight that there is large variation in the indications for aTSA in real-world surgical cases. We characterize the preoperative/baseline characteristics of those determined appropriate, inappropriate, and inconclusive based on a validated appropriateness-use criteria, and our findings. Studies evaluating the use of the algorithm with outcomes data are ongoing.

## Conclusion

Using real-world data corelated into a modified version of prior scenario-based appropriateness use criteria, approximately 18% of primary anatomic TSAs performed were classified inappropriate. The relatively large proportion of patients classified “inconclusive” may be secondary to factors such as worse glenoid morphology and/or prior rotator cuff repair, which are subjects of current debate in determining appropriateness for reverse shoulder arthroplasty or primary anatomic. The large proportion of “inconclusive” patients suggests a lack of consensus regarding anatomic versus reverse TSA candidacy. Without postoperative outcome data, no definitive conclusions can be made to determine if the use of this algorithm will ultimately improve patient outcomes after anatomic TSA. To that matter, this study seeks to only help streamline patient evaluation based on ASES high-volume surgeons' opinion and highlight there is large variation in the indications for aTSA in real-world surgical cases.
